# Examination of the Potential Moderating Role of Psychological Wellbeing in the Relationship Between Depression and Thoughts of Self-Harm in Autistic Adolescents and Adults: A Two-Year Longitudinal Study

**DOI:** 10.1007/s10803-024-06489-x

**Published:** 2024-07-30

**Authors:** Darren Hedley, Mirko Uljarević, Simon M. Bury, Alexandra Haschek, Amanda L. Richdale, Julian N. Trollor, Mark A. Stokes

**Affiliations:** 1 Olga Tennison Autism Research Centre, School of Psychology and Public Health, La Trobe University, Melbourne, VIC Australia; 2 Stanford University, Stanford, CA United States of America; 3 Department of Developmental Disability Neuropsychiatry, School of Clinical Medicine, UNSW, Sydney, NSW Australia; 4 Cooperative Research Centre for Living with Autism (Autism CRC),, Brisbane, QLD Australia; 5 Deakin University, Melbourne, VIC Australia

**Keywords:** Autism Spectrum Disorder, Adolescents, Adults, Autistic traits, Depression, Positive wellbeing, Psychological wellbeing, Self-harm, Suicide, Suicidal ideation, Wellbeing

## Abstract

**Purpose:**

Autistic people have a significantly increased risk of death by suicide relative to the general population. In non-autistic samples, psychological wellbeing has been shown to moderate the relationship between depression and suicidal thoughts and behavior. Thoughts of self-harm may provide a useful indicator of suicidal risk. In this longitudinal study we examined (a) the potential role for psychological wellbeing to moderate the relationship between depressive symptoms and thoughts of self-harm and (b) the contribution of autistic traits to thoughts of self-harm.

**Methods:**

Participants were 209 autistic adolescents and adults aged 15 to 80 years (*M*_age_ = 34.20, *SD* = 15.38 years).

**Results:**

At both baseline and 2-year follow-up, 35% of participants reported recent thoughts of self-harm. Wellbeing was associated with autistic traits (*r =* − .350 to − 0.404) and depression (*r =* − .480 to − 0.759). Thoughts of self-harm were positively associated with autistic traits and depression (*r =* .242 to 0.659), and negatively associated with wellbeing (*r =* − .287 to − 0.609). Controlling for baseline thoughts of self-harm, depression (*β* = 0.254, *p =* .001) and autistic traits (*β* = 0.162, *p =* .007) significantly predicted thoughts of self-harm at 2-year follow-up.

**Conclusion:**

Despite a lack of support for the hypothesis that wellbeing would moderate the relationship between depression and thoughts of self-harm, correlational data demonstrated significant associations between wellbeing and both depression and thoughts of self-harm. Future research considering psychological wellbeing as a potential protective factor for self-harm in autistic people is warranted.

**Supplementary Information:**

The online version contains supplementary material available at 10.1007/s10803-024-06489-x.

Suicide accounts for 1.4% of deaths worldwide, is ranked as the 14th leading cause of death across all ages, the second leading cause of death among 15 to 29-year-olds, and significantly impacts individuals, families, and society (Casey et al., 2008; GBD 2017 Causes of Death Collaborators, 2018). Findings across multiple international studies in autistic samples found that rates of self-harm and suicide behavior (including death, ideation, and attempts) are significantly elevated compared to the general population (Cassidy et al., 2014; Hedley & Uljarević, 2018; Hirvikoski et al., 2016; Huntjens et al., 2023; Kirby et al., 2019; Kõlves et al., 2021; Newell et al., 2023). Thoughts of self-harm indicate emotional distress and have been found to be associated with significant mental distress, major depression, and suicidality (Evans et al., 2004; Klonsky, 2011; Pfaff & Almeida, 2004; Pratt & Brody, 2014). Mechanisms underpinning thoughts of self-harm in autistic people may, therefore, offer insights into the high rate of suicidality within this vulnerable population.

## Risk Factors

Risk factors for self-harm and suicidality in autism that have been examined in the literature include thwarted belongingness, perceived burdensomeness (i.e., Joiner’s Interpersonal Theory of Suicide; 2005) (Moseley et al., 2023; Pelton & Cassidy, 2017; Pelton et al., 2020), increased loneliness (Hedley et al., 2018; Hedley, Uljarević, Wilmot, Hedley et al., 2018a, b), co-occurring psychopathology, and behavior problems (Jokiranta-Olkoniemi et al., 2021; McDonnell et al., 2020; for a recent and comprehensive review of the literature, see Brown et al., 2024). Co-occurring intellectual disability has been found to be associated with greater risk of suicide attempt (Hand et al., 2020) but lower risk of death by suicide (Hirvikoski et al., 2016). Several studies have examined potential associations between autistic traits and suicidality predicting a positive association, although findings are mixed (Cassidy, Bradley, Shaw et al., 2018; Hedley et al., 2018; Hunsche et al., 2020; McDonnell et al., 2020; Pelton & Cassidy, 2017; Pelton et al., 2020).

Affective disorders are by far the most significant psychiatric factors associated with self-harm and suicidality in non-autistic populations (Franklin et al., 2017; Nock et al., 2009). Similarly, the association between depression and suicidal behavior is relatively well established in studies involving autistic participants (Cassidy et al., 2014; Hedley et al., 2018a; South et al., 2020). For example, in a Finnish birth cohort study, non-affective psychoses, childhood disorders and affective and anxiety disorders explained the increased rate of intentional self-harm including suicide attempts in autistic compared to matched non-autistic controls, suggesting the presence of co-occurring psychiatric conditions can partially explain increased suicide risk in autism (Jokiranta-Olkoniemi et al., 2021). Currently, there is limited understanding of the mechanisms that link mental health conditions to suicidal behavior in the autistic population (South et al., 2020). Better understanding of factors that might moderate or mediate the relationship between affective disorders and thoughts of self-harm or suicidality is likely to be helpful for understanding, and ultimately reducing the rate of suicide deaths among the autistic population.

## Protective Factors

Studies on poor mental health, self-harm, and suicidality within the general population tend to focus on risk rather than protective factors. Specific protective factors for thoughts of self-harm or suicidality identified in studies with non-autistic samples include greater task-oriented coping, higher self-perception/competency, higher physical activity, purpose-in-life, resilience, positive relationships, life satisfaction, wellbeing, and connectedness (Bakken et al., 2024; Ki et al., 2024; Kleiman & Riskind, 2013; Sharaf et al., 2009). There are, however, few studies utilizing autistic samples. Radoeva et al. (2022) found that engagement in recreational, educational or vocational activities was associated with lower depressive symptoms in autistic adults. Furthermore, family history of depression and anxiety was associated with increased likelihood of self-harm on the Patient Health Questionnaire-9 (PHQ-9) (Kroenke & Spitzer, 2002; Kroenke et al., 2001). Similarly, increased social support and social connection may serve as a protective factor for suicidality (Hedley et al., 2017, 2018a; Mournet et al., 2023). Nonetheless, protective factors that might moderate the relationship between co-occurring mental health symptoms, thoughts of self-harm, and suicidality within the autistic population remain poorly understood.

When considering mechanisms underpinning poor mental health, psychological wellbeing is one candidate that has received attention in the non-autistic literature. Psychological wellbeing encompasses constructs such as subjective happiness, life satisfaction, psychological functioning, and self-realization (Keyes, 2005; Keyes et al., 2002; Ryff & Keyes, 1995; Tennant et al., 2007; Waterman, 1993; Westerhof & Keyes, 2010). Psychological wellbeing can be indexed by cognitive, emotional, and psychological factors, with higher levels of wellbeing associated with, for example, better coping with stress, productivity, and inter-personal relationships (Huppert, 2009; Tennant et al., 2007), including in autistic adults (Muniandy et al., 2023). Importantly, psychological wellbeing may provide a buffer against negative mental health outcomes (Wilhelm et al., 2010), including suicidality (Teismann et al., 2018; Teismann, Forkmann, Teismann et al., 2018a, b).

Several findings suggest the importance of psychological wellbeing in autistic people. Wellbeing moderated the relationship between depression and suicidal ideation in a German longitudinal study of risk and protective factors underlying mental health outcomes in non-autistic adults (Teismann et al., 2018), and there was a reduced risk of suicide attempts in clinical inpatients who experienced suicidal ideation in the presence of moderate to high levels of positive mental health (Teismann, Brailovskaia, Teismann et al., 2018a, b). In autistic adults, wellbeing was related to improved quality of life both cross-sectionally and two years later (Lawson et al., 2020), and moderated the relationship between baseline and 12-month follow-up depressive symptoms in employed autistic adults (Hedley et al., 2019). To date, the hypothesis that psychological wellbeing may moderate the relationship between depression and thoughts of self-harm or suicidality has not been studied in an autistic sample.

## Study Aims

In the present study we adopted a longitudinal design to examine the potential role of psychological wellbeing as a buffer for the relationship between depressive symptoms and thoughts of self-harm in autism. We utilized data from two longitudinal samples comprising autistic adolescents and adults. Based on research with non-autistic samples that has identified positive wellbeing as a moderator between depression and suicidal ideation (Teismann et al., 2018), we hypothesized that psychological wellbeing as measured by the Warwick-Edinburgh Mental Wellbeing Scale would buffer the association between depression and thoughts of self-harm. Given mixed findings of a significant relationship between autistic traits and self-harm or suicidality in autistic and non-autistic samples (Hedley et al., 2017, 2018a; Hochard et al., 2020; Pelton & Cassidy, 2017; Richards et al., 2019), we also examined whether autistic traits would significantly predict thoughts of self-harm when considering depression and wellbeing. We hypothesized that autistic traits would be positively associated with depression and thoughts of self-harm, negatively associated with wellbeing, and would independently predict thoughts of self-harm when controlling for wellbeing and depression.

## Methods

### Participants

The initial sample at baseline (T1) included 528 autistic people (56.3% women, 38.1% men, 5.7% non-binary) aged 15–80 years (*M* = 36.97, *SD* = 14.01), drawn from one of two longitudinal studies conducted in in Australia: the Longitudinal Study of Australian School Leavers with Autism, (SASLA, 15–25 years, *n =* 112) (Richdale et al., 2022) and the Australian Longitudinal Study of Autism in Adulthood (ALSAA, 25 + years, *n =* 416) (Arnold et al., 2019). Two-year follow-up (T2) data were available for 209 participants (48.3% women, 47.4% men, 4.3% non-binary; *n =* 83 [SASLA] and *n =* 126 [ALSAA]) aged 15–80 years (*M* = 34.20, *SD* = 15.38) who formed the sample for the present analyses.

Bootstrapped (5000 samples) (Efron & Tibshirani, 1993; Tabachnick & Fidell, 2007) *t*-tests or Chi-square analyses revealed no significant differences between included and excluded participants on the primary study variables; however, included participants were significantly older (Cohen’s *d* = 0.341) and returned marginally but significantly lower daily living skills (*d =* 0.357) compared to excluded participants. Additionally, while most males at baseline were retained at follow-up, there was a greater portion of females and individuals with a gender identity reported as “other” who did not provide follow-up data. This resulted in a significant gender difference between included and excluded participants (refer to Supplementary Table S1 for analyses comparing included and excluded participants). At T1, 45% (*n =* 94) of participants reported they were currently studying and 43.1% reported having some form of employment (*n =* 90). Participants lived with parent(s) (40.2%, *n =* 84), a partner (26.3%, *n =* 55), alone (17.2%, *n =* 36), or with others (10%; *n =* 21), with a small portion (6.2%, *n =* 13) reporting other living conditions or were missing data. Most participants were born in Australia (78.9%, *n =* 165) and primarily spoke English in the home (96.7%, *n =* 202).

### Procedure

Ethics Approval was received from relevant university ethics committees and informed consent was obtained from the participants; where participants were aged under 18 years their guardians also provided consent. Potential participants were contacted nationally (through flyers, online sites, autism organizations, education institutions, participant databases, and clinical services) (Arnold et al., 2019; Richdale et al., 2022). Following consent, participants received a link to the survey which was hosted on Qualtrics (2017); a hard copy survey was available on application. Data were collected between 2015 and 2021 via self-report questionnaires (parent report for SASLA daily living skills only), with T2 data collected approximately 2-years after T1.

#### Community Consultation

Prior to commencement of the SASLA study, community consultation was undertaken to determine research priority areas through an adult forum with members of the autism community and a national collaboration of an advisory network, researchers, and clinical health providers, including autistic individuals, parents, and peak organizations. Mental health and wellbeing were identified as critical areas of interest, and scales used to measure these constructs were included in the final survey. Local autism associations, individual parents, and young adults on the autism spectrum and a research advisory network were consulted to develop the online surveys to ensure they were relevant, valid, and accessible. Additionally, our program of research examining suicidality in autism included an autistic advisory group who were consulted and advised on the design of the present study.

### Measures

#### Daily Living Skills

Waisman Activities of Daily Living (W-ADL) (Maenner et al., 2013) is a 17- item scale designed to assess daily living skills in individuals with developmental disability, including autism, and is strongly associated with intelligence quotient (IQ) score (Maenner et al., 2013). Items are rated from 0 to 2 (range 0–34), with higher scores indicating greater independence. The W-ADL was completed by parent- (SASLA) or self-report (ALSAA). In the present study, McDonald’s omega (*ω*) = 0.866 (T1).

#### Autistic Traits

AQ-Short (Hoekstra et al., 2011) is a 28-item, self-report measure designed to assess autistic traits in individuals with an average or above IQ score (Baron-Cohen et al., 2001). Respondents answer statements on a 4-point Likert-type scale (range = 28–112), with higher scores indicating increased autism traits. In the present study, a total score was generated if no more than 10% of items were missing (Baron-Cohen et al., 2001). In the present study, *ω* = 0.862–0.875 (T1, T2).

#### Psychological Wellbeing

Warwick-Edinburgh Mental Wellbeing Scale (WEMWBS) (Tennant et al., 2007) is a 14-item scale designed to assess wellbeing including affect (e.g., optimism, cheerfulness), satisfaction with interpersonal relationships, and functioning (e.g., energy, competence, autonomy). Items load on a single factor (Tennant et al., 2007). Although no validation studies have been performed with autistic populations, WEMWBS full (Hedley et al., 2019; Hull et al., 2019) and short (Maitland et al., 2021) versions have previously been used with autistic samples, returning good reliability (e.g., Cronbach’s *α =* 0.85–0.92). The scale is completed using a 5-point Likert scale; item scores are summed to derive a total score (range = 14–70), with higher scores indicating higher levels of wellbeing. In the present study, *ω* = 0.923–0.933 (T1, T2).

#### Depression

For the SASLA sample, depressive symptoms were assessed with the Hospital Anxiety and Depression Scale depression subscale (HADS-D) (Zigmond & Snaith, 1983). HADS-D consists of 7-items assessing depressive symptoms on a 4-point Likert scale (range = 0–28); it does not include any items concerning suicidality. For the ALSAA sample HADS-D was not administered, therefore depressive symptoms were assessed with the first eight items of the Patient Health Questionnaire (henceforth, PHQ-8) (Kroenke et al., 2009). Our decision to use HADS-D depression data for SASLA participants (constituting 39.7% of the total sample) instead of PHQ-8 was aimed at reducing as much as possible the reliance on a single scale to assess both depression and thoughts of self-harm––notably, due to HADS-D not being administered to the ALSAA sample, this was not possible across the entire sample. We felt the advantages of using a separate measure for approximately 40% of the sample outweighed any possible advantages of using a single measure for depression. For analyses, depression *Z-*scores were therefore calculated for each sample and combined into a single depression score (we also provide supplementary analyses where we statistically controlled for group by including it as a covariate and utilizing PHQ-8 scores for the SASLA sample). On the PHQ-8 respondents rated 8-items regarding depressive symptoms on a 4-point Likert scale (range = 0–24) in the past two weeks. On both instruments, higher scores indicate greater depressive symptoms. Both scales have been utilized and validated with autistic samples (Arnold et al., 2020; Moseley et al., 2022; Uljarević et al., 2017). In the present study, *ω* = 0.708–0.823 (HADS-D) and 0.889–0.901 (PHQ-8; T1, T2).

#### Thoughts of Self-Harm

Thoughts of self-harm were assessed with item-9 (“Thoughts that you would be better off dead, or of hurting yourself”; range 0–3) of the PHQ-9 (Kroenke & Spitzer, 2002; Kroenke et al., 2001). This item has been found to provide an independent predictor of subsequent suicide attempt in clinical samples when controlling for PHQ-8 scores, hazard ratio = 1.91 (95% CI: 1.79, 2.04) and suicide death, hazard ratio = 1.92 [1.53, 2.41] (Simon et al., 2013). This approach (i.e., PHQ-8 as a depression measure and item 9 as an independent measure of thoughts of self-harm or suicide) has also been reported in research with autistic samples (Hedley et al., 2018; Hedley, Uljarević, Wilmot Hedley et al., 2018a, b; Radoeva et al., 2022) thereby providing additional support for the application of this approach in the present study. Higher PHQ item 9 scores indicate increased thoughts of self-harm.

### Data Cleaning and Analysis

Analyses were conducted in SPSS version 27 (IBMCorp., 2020). Across the sample (*n =* 209), missing data for T1 variables were: W-ADL = 22.5%, AQ-Short = 1.4–2.9%, WEMWBS = 1.9–2.4%, HADS-D (SASLA) = 16.9–18.1%, PHQ-9 = 3.3–4.3%. Little’s MCAR test was not significant for the analysis including W-ADL, AQ-Short, WEMWBS, PHQ-9 (item-9), *χ2*(646) = 706.330, *p =* .05, and HADS-D (SASLA), *χ2*(12) = 8.106, *p =* .777, but was for the PHQ-9, *χ2*(36) = 62.600, *p =* .004. Normality of the variables was assessed by visually inspecting P–P plots and histograms. Several variables violated the assumptions of normality, thus bootstrapping analysis with 5000 re-samples was applied (Efron & Tibshirani, 1993; Tabachnick & Fidell, 2007). Statistical significance was determined by 95% bias-corrected and accelerated confidence intervals (BCa 95% CI).

Given that overall data were not missing at random, cases with item-level missing data were excluded from descriptive and correlational analyses. Mean score replacement was applied to predictor variables for all regression analyses. All metric predictors were *z-*standardized for analyses. We first used correlations to explore associations between possible covariates (i.e., age, gender, daily living skills) and study variables. We examined changes in variables over time using bootstrapped *t-*tests for dependent samples with Cohen’s *d* for effect size. We then ran a linear multiple regression analysis to examine predictors (T1) of self-harm (T2). Baseline (T1) self-harm was included in the model.

## Results

### Descriptive Statistics

Means and standard deviation and bootstrapped paired *t-*test comparisons for study variables are provided in Table 1. No significant differences were identified between T1 and T2 for any of the study variables. At T1, 35.82% (*n =* 72; data were missing for *n =* 8 participants) participants reported thoughts of self-harm (i.e., PHQ-9 item-9 score > 0) over the last 2-weeks, and this was similar at T2, with 35.41% (*n =* 74) of participants reporting thoughts of self-harm.

**Table 1:** Descriptive (M, SD, Range)and T1–T2 bootstrapped Comparisons^1^

	T1				T2				Bootstrapped comparisons (T1–T2)			
	*n*	*M*	*SD*	Range	*n*	*M*	*SD*	Range	*t*(df)	*p-*value	BCa 95% CI	*d* [95% CI]
Daily Living	162	30.14	4.84	15–34	–	–	–	–	–	–	–	–
Autistic Traits	209	84.04	12.00	49–111	207	83.59	12.50	54–112	0.683(206)	0.502	–0.667, 1.397	0.047 [–0.089, 0.183]
Wellbeing	204	40.55	10.02	14–65	206	41.42	9.91	14–64	–1.42(200)	0.160	–2.105, 0.371	0.100 [–0.239, 0.038]
HADS-D^a^	67	5.84	3.33	0–16	83	5.29	4.01	0–14	1.248(66)	0.219	–0.309, 1.239	0.152 [–0.89, 0.393]
PHQ-8^b^	121	9.64	6.35	0–24	123	9.14	6.64	0–24	1.616(117)	0.110	–0.118, 1.664	0.149 [–0.033, 0.330]
Thoughts of Self-harm	201	0.56	0.876	0–3	209	0.54	0.866	0–3	0.625(200)	0.531	–0.065, 0.129	0.044 [–0.094, 0.182]

Note. ^1^Five thousand bootstrap samples; 95% BCa confidence intervals that do not cross zero are bolded. HADS-D: Hospital Anxiety and Depression Scale (SASLA); PHQ-8 Patient Health Questionnaire, 8 item (ALSAA); Thoughts of Self-harm: PHQ item 9

### Correlations

Bootstrapped correlation coefficients (upper panel) and confidence intervals (lower panel) between age, gender, and study variables are provided in Table 2. Age was significantly and positively correlated with daily living skills and autistic traits, with effect sizes in the small to large range (*r =* .268 to 0.517). Binary gender was significantly correlated with autistic traits (T2), although the association was small (*r*_pb_*=* 0.220). Age, gender, and daily living skills were not significantly associated with any of the other study variables, including thoughts of self-harm. Wellbeing was significantly negatively associated with autistic traits with medium effect size (*r =* − .350 to − 0.404) and depression, with large effect sizes (*r =* − .480 to − 0.759). Self-harm (T1, T2) was significantly positively associated with autistic traits and depression (*r =* .242 to 0.659), and significantly negatively associated with wellbeing (*r =* − .287 to − 0.609).

**Table 2:** Pearson’s bootstrapped correlations^1^ (Upper Panel) and BCa 95% CI (Lower Panel)

Variables	1.	2.	3.	4.	5.	6.	7.	8.	9.	10.	11.
1. Age	–	0.009	0.517***	0.268***	0.333***	–0.117	–0.027	–0.048	–0.047	0.026	–0.018
2. Gender (Binary)^2^	–0.178, 0.150	–	0.132	0.139	0.220*	–0.043	–0.016	0.020	0.044	0.049	0.105
3. Daily Living	**0.413**,** 0.606**	–0.036, 0.277	–	0.074	0.146	0.044	0.123	–0.043	–0.155	0.005	–0.107
4. Autistic Traits (T1)	**0.109**,** 0.419**	–0.021, 0.305	–0.112, 0.249	–	0.786***	–0.355***	–0.350***	0.214**	0.291***	0.242**	0.251**
5. Autistic Traits (T2)	**0.183**,** 0.483**	**0.057**,** 0.380**	–0.037, 0.321	**0.703**,** 0.855**	–	–0.395***	–0.404***	0.246**	0.366***	0.247**	0.253**
6. Wellbeing (T1)	–0.272, 0.038	–0.202, 0.116	–0.126, 0.216	**–0.489**,** –0.209**	**–0.513**,** –0.259**	–	0.562***	–0.617***	–0.483***	–0.507***	–0.287***
7. Wellbeing (T2)	–0.187, 0.130	–0.172, 0.145	–0.026, 0.276	**–0.490**,** –0.197**	**–0.535**,** –0.258**	**0.420**,** 0.685**	–	–0.480***	–0.759***	–0.371***	–0.609***
8. Depression (T1)^3^	–0.206, 0.115	–0.150, 0.194	–0.210, 0.116	**0.067**,** 0.362**	**0.097**,** 0.385**	**–0.705**,** –0.515**	**–0.602**,** –0.342**	–	0.624***	0.518***	0.422***
9. Depression (T2)^3^	–0.201, 0.111	–0.127, 0.212	–0.323, 0.009	**0.135**,** 0.440**	**0.217**,** 0.501**	**–0.590**,** –0.361**	**–0.825**,** –0.673**	**0.505**,** 0.726**	–	0.429***	0.659***
10. Self-harm (T1)	–0.123, 0.176	–0.117, 0.204	–0.125, 0.128	**0.072**,** 0.404**	**0.097**,** 0.386**	**–0.627**,** –0.364**	**–0.512**,** –0.217**	**0.392**,** 0.630**	**0.264**,** 0.572**	–	0.590***
11. Self-harm (T2)	–0.153, 0.132	–0.059, 0.264	–0.281, 0.063	**0.070**,** 0.415**	**0.053**,** 0.422**	**–0.424**,** –0.136**	**–0.712**,** –0.481**	**0.276**,** 0.559**	**0.561**,** 0.739**	**0.430**,** 0.730**	–

Note.^1^Five thousand bootstrap samples; 95% BCa confidence intervals that do not cross zero are bolded. Point-biserial correlations (*r*_pb_) were applied where analyses include continuous and dichotomous variables. ^2^Bootstrapped point-biserial correlation, exclude non-binary gender (*n =* 9). ^3^Depression scores are derived from Hospital Anxiety and Depression Scale (HADS-D; SASLA) and Patient Health Questionnaire (PHQ-8; ALSAA).

*p < .05

**p < .01

***p < .001

### Linear Multiple Regression Model

Results of the linear multiple regression model predicting thoughts of self-harm are provided in Table 3. Because preliminary screening analyses showed age, gender and daily living skills were not related to self-harm (T1 or T2), these were not proceeded further with. Variables (T1) entered into the model were self-harm, autistic traits, psychological wellbeing, depression, and the two-way-interaction between depression and psychological wellbeing, the dependent variable was self-harm at T2. Contribution of variables to the model were examined using standardized beta coefficients (*β*) and semi-partial correlations (*sr*). Standardized beta coefficients (*β*) and semi-partial correlations (*sr*) were calculated without bootstrapping. For consistency, non-bootstrapped *p*-values are provided in text and bootstrapped *p-*values are reported in the tables. We also ran models where we (a) statistically controlled for potential group differences by including group as a covariate (Supplementary Table S2) and (b) examined a model where we replaced HADS-D depression scores for the SASLA sample thereby improving data harmonization (Supplementary Table S3). Overall, these models were similar to the original model with no significant differences between models in terms of identified predictors.

**Table 3:** Bootstrapped Hierarchical Linear regression models with T1 variables Predicting thoughts of Self-harm at T2

	b	SEB	β	*p*-value^b^	BCa 95% CI^c^
**Constant**	–0.023	0.054	–	0.672	[–0.126, 0.084]
Thoughts of Self-harm (T1)	0.504	0.063	0.517	**< 0.001**	**[0.362**,** 0.661]**
Autistic Traits^a^	0.156	0.058	0.162	**0.007**	**[0.045**,** 0.264]**
Wellbeing^a^	0.091	0.073	0.093	0.195	[–0.044, 0.228]
Depression^a^	0.258	0.075	0.254	**< 0.001**	**[0.108**,** 0.416]**
Depression × Wellbeing^a^	0.116	0.061	0.115	0.067	[–0.008, 0.236]
Model	*R*^*2*^ = 0.400, *F*(5, 200) = 26.040, *p <* .001				

Note. ^a^*z*-score used in analysis. ^b^5000 samples bootstrapped *p-*value. ^c^BCa 95% confidence intervals that do not cross zero are bolded

The full model accounted for 40.0% of the variance in T2 thoughts of self-harm, *F*(5, 200) = 26.040, *p* < .001. Significant predictors of thoughts of self-harm at follow-up (T2) included baseline self-harm (T1), autistic traits (T1), and depression (T1). The b-weights revealed that for each unit increase in baseline thoughts of self-harm (T1), self-harm at follow-up (T2) increased by 0.504 units, for each unit increase in autistic traits, thoughts of self-harm (T2) increased by 0.156 units, and for each unit increase in depression, self-harm (T2) increased by 0.258 units. However, neither wellbeing nor the Depression × Wellbeing interaction significantly predicted T2 thoughts of self-harm; for each unit increase in wellbeing, self-harm (T2) increased by 0.091 units. Overall, baseline self-harm (T1) made the largest significant contribution to self-harm at follow-up (T2) (*β* = 0.517, *sr* = 0.498, *p* < .001), followed by depression (*β* = 0.254, *sr* = 0.239, *p* = .001) and autistic traits (*β* = 0.162, *sr* = 0.191, *p* = .007). Wellbeing (*β* = 0.091, *sr* = 0.089, *p* = .195) and the interaction (*β* = 0.115, *sr* = 0.135, *p* = .067) made small and statistically non-significant contributions to the model. Given the two-way-interaction of wellbeing and depression did not significantly predict thoughts of self-harm, there was no evidence that psychological wellbeing moderates the effect of depression on thoughts of self-harm.

## Discussion

In this study, we evaluated the hypothesis that psychological wellbeing acts as a buffer between depression and thoughts of self-harm. We also examined whether autistic traits would be associated with depression and self-harm. Our data were drawn from a longitudinal sample of autistic adolescents and adults. We analyzed baseline (T1) data as well as follow-up (T2) data which was collected two years following T1 data collection. Consistent with our hypothesis predicting relationships between wellbeing, depression, and self-harm, we found that wellbeing was negatively associated with both depression and self-harm. However, when controlling for the effects of depression on self-harm, the association between wellbeing and self-harm was no longer statistically significant. Thus, our primary hypothesis that psychological wellbeing would moderate the relationship between depression and thoughts of self-harm was not supported. Our regression model identified autistic traits and depression scores as the only statistically significant predictors of thoughts of self-harm scores two years later, controlling for baseline thoughts of self-harm.

Autistic traits were significantly negatively correlated with wellbeing, and positively associated with depression and thoughts of self-harm. Moreover, when entered in the regression analysis, autistic traits emerged as a significant predictor of thoughts of self-harm. This finding contrasts with research in other autistic samples where autistic traits have not been found to reliably predict thoughts of self-harm or suicidality (Cassidy, Bradley, Shaw et al., 2018; Hedley et al., 2018; Hedley et al., 2017; Hedley, Uljarević, Wilmot, Hedley et al., 2018a, b). In Cassidy, Bradley, Shaw et al.’s (2018) study, suicide risk was assessed with the Suicide Behaviors Questionnaire-Revised (SBQ-R) (Osman et al., 2001), a 4-item self-report questionnaire that assesses suicidal thoughts and behavior in the past 12 months. Thus, suicidality differed from the construct of self-harm assessed with the PHQ-9 in our study, which might explain differences in findings. However, this does not entirely explain our findings as, consistent with our approach, Hedley and colleagues (Hedley et al., 2017, 2018a; Hedley, Uljarević, Wilmot, Hedley et al., 2018a, b) utilized the PHQ-9 to provide an indicator of self-harm/suicidality.

It is interesting to note that research in non-autistic samples has found significant relationships between autistic traits and self-harm or suicidality (Cassidy, Bradley, Shaw et al., 2018; Cassidy et al., 2020; Hedley et al., 2021; Hochard et al., 2020; Richards et al., 2019). Different findings between autistic and non-autistic samples may reflect characterization of the underlying construct of autistic traits in clinical (i.e., enriched) and general population samples––although autistic traits are thought to reflect a dimensional structure normally distributed within the general population, the distribution of autistic traits in clinically enriched samples indicates a dual distribution model suggesting underlying categorical differences with respect to the extent to which autistic traits are present in autistic and non-autistic populations (Abu-Akel et al., 2019). Second, the 50-item AQ may function differently for autistic and non-autistic groups, although the AQ-28 addresses some of these limitations through removal of poorly performing items (Agelink van Rentergem et al., 2019). Differences in measures across studies may also affect relationships with autistic traits. For example, Hochard et al. (2020) used the four-item Depression Severity Index-Suicide Subscale (DSI-SS) in their study, whereas we used a single item on thoughts of self-harm. The lack of association between autistic traits and depressive symptoms that has been reported in some studies (e.g., Cai et al., 2023), or that the connection between autism and depression may occur through a third factor such as mastery (van Heijst et al., 2020), may also impact any relationship between autistic traits and thoughts of self-harm. Further research into potential relationships between autistic traits and mental health and wellbeing, particularly self-harm and suicidality, is required. Indeed, if there is a relationship between autistic traits and self-harm or suicidality within the autistic population, better understanding of the mechanisms underlying these relationships may be important for the development of effective suicide intervention and prevention strategies.

Our results are inconsistent with findings from non-autistic samples which offer support for the role of wellbeing as a buffer between depression and suicidality (i.e., Siegmann et al., 2018; Teismann et al., 2018; Teismann, Forkmann, Teismann et al., 2018a, b). In our sample of autistic adolescents and adults, one possibility is that wellbeing may not be salient to psychological functioning in the same way as in a non-autistic population. Other factors, such as social support, may more reliably offset the impact of depression on self-harm in autistic people. In autistic samples, satisfaction with social support has been found to mediate the relationship between the number of supports the individual had access to and both depression and loneliness (Hedley et al., 2018). Social support in the form of perceived availability of tangible supports was found to be indirectly associated with self-harm (PHQ-9 item 9) through depression, suggesting it may act as a protective factor for thoughts of self-harm (Hedley et al., 2017). In non-autistic samples, depression mediated the relationship between social support and suicidal ideation, supporting the hypothesis that better social support may be particularly salient to depressed individuals who are susceptible to thoughts of self-harm or suicidality (Kim et al., 2021).

It is important to acknowledge methodological differences between the present study and previous research. While we assessed wellbeing with the WEMWBS (Tennant et al., 2007), other studies have utilized different measures, for example, the Positive Mental Health Scale (Lukat et al., 2016) used by Teismann and colleagues (Teismann et al., 2018; Teismann, Forkmann, Teismann et al., 2018a, b). The two scales differ such that the WEMWBS assesses participants’ current experience of psychological wellbeing (e.g., “I’ve been feeling loved”) whereas the PMH-scale is more attitudinal (e.g., “I feel that I am actually well equipped to deal with life and its difficulties”). It may be that attitudes to psychological wellbeing are more enduring and impactful on self-harm, than positive feelings. This possibility warrants further exploration.

We did not identify any change in mental health and wellbeing scores over the two-year period when data were collected, suggesting stability in these factors over time; this stability in mental health over extended periods of time is consistent with previous studies in different autistic samples (Hedley et al., 2019). Similarly, in other studies using different subsets of the SASLA dataset used here, there was also no change in depression or mental wellbeing (Muniandy et al., 2023; Richdale et al., 2023a), quality of life (Lawson et al., 2020), or fatigue (Richdale et al., 2023) over a 2-year period. Additionally, mental wellbeing was associated with improved quality of life cross-sectionally and at 2-year follow-up in autistic adults, but like the current study, T1 quality of life was the only significant predictor of quality of life 2-years later (Lawson et al., 2020). These findings suggest that, at least for the autistic adolescents and adults in these data sets, mental health, wellbeing, and related variables do not change over time and that environmental impacts such as lack of supports or access to treatment may be responsible.

Without focused intervention to address poor mental health and wellbeing outcomes, things are unlikely to improve for autistic adults. Indeed, the study by Hedley et al. (2019) demonstrated that even transitioning to well-paid employment failed to significantly budge health and wellbeing scores in autistic adults. The absence of psychological wellbeing as a moderator on the relationship between depression and thoughts of self-harm might have been affected by this lack of variability in scores over time. Most likely, the strong relationship between depression and thoughts of self-harm may simply override other, smaller effects. As noted by others (Culpin et al., 2018), shifting depression is likely critical to positively impacting the high rate of self-harm and suicidality within the autistic population.

There are other variables associated with depression that may, directly or indirectly through depression, influence self-harm and suicidality in autistic adults. In other samples, disengagement coping style (Muniandy et al., 2023), social wellbeing (Richdale et al., 2023), insomnia (Richdale et al., 2023; Richdale, Lawson, Richdale et al., 2023a, b) and specific traits commonly co-occurring with autism including sensory sensitivities, autonomic arousal, and intolerance of uncertainty (Richdale, Lawson, Richdale et al., 2023a, b) influenced depressive symptoms. Indeed, the relationship between insomnia and suicidality in autism remains unexplored, but in non-autistic samples it is an independent predictor of suicidality (Hochard et al., 2020; Lin et al., 2018). Autistic adults report high rates of insomnia and sleep difficulties (Morgan et al., 2020), suggesting that sleep is another fruitful area for further exploration. Overall, this supports the complexity of factors that may lead to thoughts of self-harm and suicidal behavior in autistic individuals.

## Strengths and Limitations

Our study was strengthened by using longitudinal data collected over two years. This goes some way toward addressing limitations associated with the high prevalence of cross-sectional studies within autism and suicide literature. Our sample also represented a relatively large and diverse in age group of autistic people. However, sample size was nonetheless smaller compared to similar studies in the general population, which may have affected our power to detect significant effects. Furthermore, there was a significant drop-out rate (39.58%) between baseline and 2-year follow-up, although our analysis revealed no significant differences between the two time-points for scores on the primary study variables. We acknowledge limitations associated with the use of a relatively broad construction of wellbeing which incorporates a range of constructs (e.g., subjective happiness, life satisfaction, psychological functioning, and self-realization). Although confirmatory factor analysis supports a single factor for the WEMWBS (Tennant et al., 2007), potential overlap among subcomponents of wellbeing, depression, and self-harm may have affected our results and the lack of support for the primary hypothesis. Future research that examines the underlying conceptual model by addressing the potential overlap of these constructs is required. Last, we were limited by the availability of only a single self-report item to assess thoughts of self-harm. The use of a single item limits our ability to assess internal consistency and may lead to more error than scales based on multiple correlated items. For the ALSAA sample, the item was drawn from the same measure as depression scores (i.e., PHQ-9).

## Future Directions

Despite the utility of PHQ-9 as a predictor of suicidality (Kim et al., 2021) and previous use of item 9 for this purpose in research involving autistic and non-autistic samples (Hedley et al., 2018; Hedley, Uljarević, Wilmot Hedley et al., 2018a, b; Radoeva et al., 2022; Simon et al., 2013), thereby supporting its use in the present study, future studies would benefit from more extensive assessment of suicidality, independent of depression measures, and ideally including assessment of previous suicide attempts. Future research should also consider a broader range of risk and protective factors that might underlie the high rate of self-harm and suicidality within the autistic population. In doing so, it will be important to consider factors that are both evident in non-autistic groups (e.g., mental health challenges, social support) as well as those that might be more salient within autistic samples (e.g., camouflaging, unmet support needs) (Cassidy et al., 2018, 2020). We hope that our findings reported here can guide future research into positive mental health factors that might be protective against the high rate of self-harm and suicidality in autism.

## Conclusion

In this study we did not find support for the hypothesis that psychological wellbeing buffered the relationship between depression and thoughts of self-harm in autistic adolescents and adults. However, our correlational data demonstrated significant associations between wellbeing and both depression and thoughts of self-harm indicating that further research into the potential role of psychological wellbeing in offsetting negative psychopathology may be justified. Our identification of baseline autistic traits as a potential predictor of thoughts of self-harm at 2-year follow-up within autistic samples is novel and requires further investigation to identify underlying mechanisms and may be useful in informing intervention and prevention strategies. This should include a non-autistic group where model invariance can be examined across groups. Stability of health and wellbeing trajectories suggests interventions may need to target autistic people at a young age (e.g., in childhood) (Culpin et al., 2018), before these trajectories become ingrained and increasingly difficult to shift.

## Electronic Supplementary Material

Below is the link to the electronic supplementary material.

Supplementary Table S1

Supplementary Table S2

Supplementary Table S3

## Data Availability

This study was pre-registered on the Open Science Foundation (OSF) website on November 23, 2020, prior to receiving raw data files (10.17605/OSF.IO/DHNW8). These data are available from the Autism CRC under license and so cannot be made freely available. Requests for access to these data should be made to the Autism CRC.

## References

[CR1] Abu-Akel, A., Allison, C., Baron-Cohen, S., & Heinke, D. (2019). The distribution of autistic traits across the autism spectrum: Evidence for discontinuous dimensional subpopulations underlying the autism continuum. *Molecular Autism*, *10*, e24. 10.1186/s13229-019-0275-3. 10.1186/s13229-019-0275-3 PMC6537408 31149329

[CR3] Arnold, S. R. C., Foley, K. R., Hwang, Y. I., Richdale, A. L., Uljarevic, M., Lawson, L. P., Cai, R. Y., Falkmer, T., Falkmer, M., Lennox, N. G., Urbanowicz, A., & Trollor, J. (2019). Cohort profile: The Australian longitudinal study of adults with autism (ALSAA). *British Medical Journal Open*, *9*(12), e030798. 10.1136/bmjopen-2019-030798. 10.1136/bmjopen-2019-030798 PMC6924702 31806608

[CR4] Arnold, S. R. C., Uljarević, M., Hwang, Y. I., Richdale, A. L., Trollor, J. N., & Lawson, L. P. (2020). Psychometric properties of the Patient Health Questionaire-9 (PHQ-9) in autistic adults. *Journal of Autism and Developmental Disorders*, *50*(6), 2217–2225. 10.1007/s10803-019-03947-9. 30847710 10.1007/s10803-019-03947-9

[CR5] Bakken, V., Lydersen, S., Skokauskas, N., Sund, A. M., & Kaasbøll, J. (2024). Protective factors for suicidal ideation: A prospective study from adolescence to adulthood. *European Child & Adolescent Psychiatry*, *2024*. 10.1007/s00787-024-02379-w. 10.1007/s00787-024-02379-w PMC11424721 38356041

[CR6] Baron-Cohen, S., Wheelwright, S., Skinner, R., Martin, J., & Clubley, E. (2001). The autism-spectrum quotient (AQ): Evidence from Asperger syndrome/high-functioning autism, males and females, scientists and mathematicians. *Journal of Autism and Developmental Disorders*, *31*(1), 5–17. 10.1023/a:1005653411471. 11439754 10.1023/a:1005653411471

[CR7] Brown, C. M., Newell, V., Sahin, E., & Hedley, D. (2024). Updated systematic review of suicide in autism: 2018–2024. Manuscript submitted for publication.

[CR8] Cai, R. Y., Love, A., Robinson, A., & Gibbs, V. (2023). The inter-relationship of emotion regulation, self-compassion, and mental health in autistic adults. *Autism in Adulthood*, *5*(3), 335–342. 10.1089/aut.2022.0068. 37663445 10.1089/aut.2022.0068 PMC10468559

[CR9] Casey, P., Dunn, G., Kelly, B. D., Lehtinen, V., Dalgard, O. S., Dowrick, C., & Ayuso-Mateos, J. L. (2008). The prevalence of suicidal ideation in the general population: Results from the outcome of Depression International Network (ODIN) study. *Social Psychiatry Psychiatric Epidemiology*, *43*(4), 299–304. 10.1007/s00127-008-0313-5. 18264810 10.1007/s00127-008-0313-5

[CR11] Cassidy, S., Bradley, P., Robinson, J., Allison, C., McHugh, M., & Baron-Cohen, S. (2014). Suicidal ideation and suicide plans or attempts in adults with Asperger’s syndrome attending a specialist diagnostic clinic: A clinical cohort study. *Lancet Psychiatry*, *1*(2), 142–147. 10.1016/s2215-0366(14)70248-2. 26360578 10.1016/S2215-0366(14)70248-2

[CR10] Cassidy, S., Bradley, L., Shaw, R., & Baron-Cohen, S. (2018). Risk markers for suicidality in autistic adults. *Molecular Autism*, *9*, e42. 10.1186/s13229-018-0226-4. 10.1186/s13229-018-0226-4 PMC6069847 30083306

[CR12] Cassidy, S., Gould, K., Townsend, E., Pelton, M., Robertson, A. E., & Rodgers, J. (2020). Is camouflaging autistic traits associated with suicidal thoughts and behaviours? Expanding the interpersonal psychological theory of suicide in an undergraduate student sample. *Journal of Autism and Developmental Disorders*, *50*, 3638–3648. 10.1007/s10803-019-04323-3. 31820344 10.1007/s10803-019-04323-3 PMC7502035

[CR13] Culpin, I., Mars, B., Pearson, R. M., Golding, J., Heron, J., Bubak, I., Carpenter, P., Magnusson, C., Gunnell, D., & Rai, D. (2018). Autistic traits and suicidal thoughts, plans, and self-harm in late adolescence: Population-based cohort study. *Journal of the American Academy of Child & Adolescent Psychiatry*, *57*(5), 313–320e316. 10.1016/j.jaac.2018.01.023. 29706160 10.1016/j.jaac.2018.01.023 PMC5942156

[CR14] Efron, B., & Tibshirani, R. (1993). *An introduction to the bootstrap*. Chapman & Hall/CRC.

[CR15] Evans, E., Hawton, K., & Rodham, K. (2004). Factors associated with suicidal phenomena in adolescents: A systematic review of population-based studies. *Clinical Psychology Review*, *24*(8), 957–979. 10.1016/j.cpr.2004.04.005. 15533280 10.1016/j.cpr.2004.04.005

[CR16] Franklin, J. C., Ribeiro, J. D., Fox, K. R., Bentley, K. H., Kleiman, E. M., Huang, X., Musacchio, K. M., Jaroszewski, A. C., Chang, B. P., & Nock, M. K. (2017). Risk factors for suicidal thoughts and behaviors: A meta-analysis of 50 years of research. *Psychological Bulletin*, *143*(2), 187–232. 10.1037/bul0000084. 27841450 10.1037/bul0000084

[CR17] GBD 2017 Causes of Death Collaborators. (2018). Global, regional, and national age-sex-specific mortality for 282 causes of death in 195 countries and territories, 1980–2017: A systematic analysis for the global burden of Disease Study 2017. *Lancet*, *392*(10159), 1736–1788. 10.1016/s0140-6736(18)32203-7. 30496103 10.1016/S0140-6736(18)32203-7 PMC6227606

[CR18] Hand, B. N., Benevides, T. W., & Carretta, H. J. (2020). Suicidal ideation and self-inflicted injury in Medicare enrolled autistic adults with and without co-occurring intellectual disability. *Journal of Autism and Developmental Disorders*, *50*(10), 3489–3495. 10.1007/s10803-019-04345-x. 31858322 10.1007/s10803-019-04345-x

[CR19] Hedley, D., & Uljarević, M. (2018). Systematic review of suicide in Autism Spectrum Disorder: Current trends and implications. *Current Developmental Disorders Reports*, *5*, 65–76. 10.1007/s40474-018-0133-6.

[CR23] Hedley, D., Uljarević, M., Wilmot, M., Richdale, A., & Dissanayake, C. (2017). Social support, depression and suicidal ideation in adults with Autism Spectrum Disorder. *Journal of Autism and Developmental Disorders*, *47*, 3669–3677. 10.1007/s10803-017-3274-2. 28861661 10.1007/s10803-017-3274-2

[CR22] Hedley, D., Uljarević, M., Foley, K. R., Richdale, A., & Trollor, J. (2018a). Risk and protective factors underlying suicidal ideation in Autism Spectrum Disorder. *Depression and Anxiety*, *35*, 648–657. https://doi.org/10.1002/da.22759. 10.1002/da.22759 29659141

[CR24] Hedley, D., Uljarević, M., Wilmot, M., Richdale, A., & Dissanayake, C. (2018b). Understanding depression and thoughts of self-harm in autism: A potential mechanism involving loneliness. *Research in Autism Spectrum Disorders*, *46*, 1–7. 10.1016/j.rasd.2017.11.003.

[CR20] Hedley, D., Uljarević, M., Bury, S. M., & Dissanayake, C. (2019). Predictors of mental health and well-being in employed adults with autism spectrum disorder at 12-month follow-up. *Autism Research*, *12*, 482–494. 10.1002/aur.2064. 30675764 10.1002/aur.2064

[CR21] Hedley, D., Uljarević, M., Cai, R. Y., Bury, S. M., Stokes, M. A., & Evans, D. W. (2021). Domains of the autism phenotype, cognitive control, and rumination as transdiagnostic predictors of DSM-5 suicide risk. *PLoS One*, *16*(1), e0245562. 10.1371/journal.pone.0245562. 33482664 10.1371/journal.pone.0245562 PMC7822649

[CR25] Hirvikoski, T., Mittendorfer-Rutz, E., Boman, M., Larsson, H., Lichtenstein, P., & Bölte, S. (2016). Premature mortality in autism spectrum disorder. *The British Journal of Psychiatry*, *208*(3), 232–238. 10.1192/bjp.bp.114.160192. 26541693 10.1192/bjp.bp.114.160192

[CR26] Hochard, K. D., Pendrous, R., Mari, T., & Flynn, S. (2020). Examining the relationship between autism traits and sleep duration as predictors of suicidality. *Journal of Autism and Developmental Disorders*, *50*(10), 3575–3584. 10.1007/s10803-020-04405-7. 32086693 10.1007/s10803-020-04405-7 PMC7502050

[CR27] Hoekstra, R. A., Vinkhuyzen, A. A., Wheelwright, S., Bartels, M., Boomsma, D. I., Baron-Cohen, S., Posthuma, D., & van der Sluis, S. (2011). The construction and validation of an abridged version of the autism-spectrum quotient (AQ-Short). *Journal of Autism and Developmental Disorders*, *41*(5), 589–596. 10.1007/s10803-010-1073-0. 20697795 10.1007/s10803-010-1073-0 PMC3076581

[CR28] Hull, L., Mandy, W., Lai, M. C., Baron-Cohen, S., Allison, C., Smith, P., & Petrides, K. V. (2019). Development and validation of the camouflaging autistic traits questionnaire (CAT-Q). *Journal of Autism and Developmental Disorders*, *49*(3), 819–833. 10.1007/s10803-018-3792-6. 30361940 10.1007/s10803-018-3792-6 PMC6394586

[CR29] Hunsche, M. C., Saqui, S., Mirenda, P., Zaidman-Zait, A., Bennett, T., Duku, E., Elsabbagh, M., Georgiades, S., Smith, I. M., Szatmari, P., Ungar, W. J., Vaillancourt, T., Waddell, C., Zwaigenbaum, L., & Kerns, C. M. (2020). Parent-reported rates and clinical correlates of suicidality in children with Autism Spectrum disorder: A longitudinal study. *Journal of Autism and Developmental Disorders*, *50*(10), 3496–3509. 10.1007/s10803-020-04373-y. 32034647 10.1007/s10803-020-04373-y

[CR30] Huntjens, A., Landlust, A., Wissenburg, S., & van der Gaag, M. (2023). The prevalence of suicidal behavior in Autism Spectrum Disorder. *Crisis*. 10.1027/0227-5910/a000922. 37668055 10.1027/0227-5910/a000922

[CR31] Huppert, F. A. (2009). Psychological well-being: Evidence regarding its causes and consequences. *Applied Psychology: Health and Well-Being*, *1*(2), 137–164. 10.1111/j.1758-0854.2009.01008.x.

[CR32] IBMCorp (2020). *IBM SPSS statistics for windows (Version 27.0) [Computer Program]*. In IBM Corp.

[CR33] Joiner, T. E. (2005). *Why people die by suicide*. Harvard University Press.

[CR34] Jokiranta-Olkoniemi, E., Gyllenberg, D., Sucksdorff, D., Suominen, A., Kronström, K., Chudal, R., & Sourander, A. (2021). Risk for premature mortality and intentional self-harm in Autism Spectrum disorders. *Journal of Autism and Developmental Disorders*. 10.1007/s10803-020-04768-x. 10.1007/s10803-020-04768-x PMC8349316 33140146

[CR35] Keyes, C. L. (2005). Mental illness and/or mental health? Investigating axioms of the complete state model of health. *Journal of Consulting and Clinical Psychology*, *73*(3), 539–548. 10.1037/0022-006x.73.3.539. 15982151 10.1037/0022-006X.73.3.539

[CR36] Keyes, C. L., Shmotkin, D., & Ryff, C. D. (2002). Optimizing well-being: The empirical encounter of two traditions. *Journal of Personality and Social Psychology*, *82*(6), 1007–1022. 10.1037/0022-3514.82.6.1007. 12051575

[CR37] Ki, M., Lapierre, S., Gim, B., Hwang, M., Kang, M., Dargis, L., Jung, M., Koh, E. J., & Mishara, B. (2024). A systematic review of psychosocial protective factors against suicide and suicidality among older adults. *International Psychogeriatrics*, *36*(5), 346–370. 10.1017/S104161022300443X. 38305360 10.1017/S104161022300443X

[CR38] Kim, S., Lee, H-K., & Lee, K. (2021a). Which PHQ-9 items can effectively screen for suicide? Machine learning approaches. *International Journal of Environmental Research and Public Health*, *18*(7), 3339. 10.3390/ijerph18073339. 33804879 10.3390/ijerph18073339 PMC8036742

[CR39] Kim, Y. J., Quinn, C. R., & Moon, S. S. (2021b). Buffering effects of social support and parental monitoring on suicide. *Health and Social Work*, *46*(1), 42–50. 10.1093/hsw/hlaa037. 33822052 10.1093/hsw/hlaa037

[CR40] Kirby, A. V., Bakian, A. V., Zhang, Y., Bilder, D. A., Keeshin, B. R., & Coon, H. (2019). A 20-year study of suicide death in a statewide autism population. *Autism Research*, *12*(4), 658–666. 10.1002/aur.2076. 30663277 10.1002/aur.2076 PMC6457664

[CR41] Kleiman, E. M., & Riskind, J. H. (2013). Utilized social support and self-esteem mediate the relationship between perceived social support and suicide ideation. *Crisis*, *34*(1), 42–49. 10.1027/0227-5910/a000159. 22846448 10.1027/0227-5910/a000159

[CR42] Klonsky, E. D. (2011). Non-suicidal self-injury in United States adults: Prevalence, sociodemographics, topography and functions. *Psychological Medicine*, *41*(9), 1981–1986. 10.1017/S0033291710002497. 21208494 10.1017/S0033291710002497

[CR43] Kõlves, K., Fitzgerald, C., Nordentoft, M., Wood, S. J., & Erlangsen, A. (2021). Assessment of suicidal behaviors among individuals with Autism Spectrum Disorder in Denmark. *JAMA Network Open*, *4*(1), e2033565. 10.1001/jamanetworkopen.2020.33565. 33433599 10.1001/jamanetworkopen.2020.33565 PMC12578491

[CR44] Kroenke, K., & Spitzer, R. L. (2002). The PHQ-9: A new depression diagnostic and severity measure [doi:10.3928/0048-5713-20020901-06]. *Psychiatric Annals*, *32*, 509–515. 10.3928/0048-5713-20020901-06.

[CR45] Kroenke, K., Spitzer, R. L., & Williams, J. B. (2001). The PHQ-9: Validity of a brief depression severity measure. *Journal of General Internal Medicine*, *16*(9), 606–613. 10.1046/j.1525-1497.2001.016009606.x. 11556941 10.1046/j.1525-1497.2001.016009606.x PMC1495268

[CR46] Kroenke, K., Strine, T. W., Spitzer, R. L., Williams, J. B., Berry, J. T., & Mokdad, A. H. (2009). The PHQ-8 as a measure of current depression in the general population. *Journal of Affective Disorders*, *114*(1–3), 163–173. 10.1016/j.jad.2008.06.026. 18752852 10.1016/j.jad.2008.06.026

[CR47] Lawson, L. P., Richdale, A. L., Haschek, A., Flower, R. L., Vartuli, J., Arnold, S. R., & Trollor, J. N. (2020). Cross-sectional and longitudinal predictors of quality of life in autistic individuals from adolescence to adulthood: The role of mental health and sleep quality. *Autism*, *24*(4), 954–967. 10.1177/1362361320908107. 32169010 10.1177/1362361320908107

[CR48] Lin, H. T., Lai, C. H., Perng, H. J., Chung, C. H., Wang, C. C., Chen, W. L., & Chien, W. C. (2018). Insomnia as an independent predictor of suicide attempts: A nationwide population-based retrospective cohort study. *Bmc Psychiatry*, *18*(1), 117. 10.1186/s12888-018-1702-2. 29716570 10.1186/s12888-018-1702-2 PMC5930777

[CR49] Lukat, J., Margraf, J., Lutz, R., van der Veld, W. M., & Becker, E. S. (2016). Psychometric properties of the Positive Mental Health Scale (PMH-scale). *BMC Psychology*, *4*(1), 8. 10.1186/s40359-016-0111-x. 26865173 10.1186/s40359-016-0111-x PMC4748628

[CR50] Maenner, M. J., Smith, L. E., Hong, J., Makuch, R., Greenberg, J. S., & Mailick, M. R. (2013). Evaluation of an activities of daily living scale for adolescents and adults with developmental disabilities. *Disability and Health Journal*, *6*(1), 8–17. 10.1016/j.dhjo.2012.08.005. 23260606 10.1016/j.dhjo.2012.08.005 PMC3531884

[CR51] Maitland, C. A., Rhodes, S., O’Hare, A., & Stewart, M. E. (2021). Social identities and mental well-being in autistic adults. *Autism*, *25*(6), 1771–1783. 10.1177/13623613211004328. 34011188 10.1177/13623613211004328 PMC8323333

[CR52] McDonnell, C. G., DeLucia, E. A., Hayden, E. P., Anagnostou, E., Nicolson, R., Kelley, E., Georgiades, S., Liu, X., & Stevenson, R. A. (2020). An exploratory analysis of predictors of youth suicide-related behaviors in Autism Spectrum Disorder: Implications for prevention science. *Journal of Autism and Developmental Disorders*, *50*(10), 3531–3544. 10.1007/s10803-019-04320-6. 31820342 10.1007/s10803-019-04320-6

[CR53] Morgan, B., Nageye, F., Masi, G., & Cortese, S. (2020). Sleep in adults with Autism Spectrum disorder: A systematic review and meta-analysis of subjective and objective studies. *Sleep Medicine*, *65*, 113–120. 10.1016/j.sleep.2019.07.019. 31739229 10.1016/j.sleep.2019.07.019

[CR54] Moseley, R. L., Gregory, N. J., Smith, P., Allison, C., Cassidy, S., & Baron-Cohen, S. (2022). The relevance of the interpersonal theory of suicide for predicting past-year and lifetime suicidality in autistic adults. *Molecular Autism*, *13*(1), 14. 10.1186/s13229-022-00495-5. 35313974 10.1186/s13229-022-00495-5 PMC8935684

[CR55] Moseley, R. L., Gregory, N. J., Smith, P., Allison, C., Cassidy, S., & Baron-Cohen, S. (2023). Potential mechanisms underlying suicidality in autistic people with attention Deficit/Hyperactivity disorder: Testing hypotheses from the interpersonal theory of suicide. *Autism in Adulthood*. 10.1089/aut.2022.0042. 10.1089/aut.2022.0042 PMC10902282 38435325

[CR56] Mournet, A. M., Wilkinson, E., Bal, V. H., & Kleiman, E. M. (2023). A systematic review of predictors of suicidal thoughts and behaviors among autistic adults: Making the case for the role of social connection as a protective factor. *Clinical Psychology Review*, *99*, e102235. 10.1016/j.cpr.2022.102235. 10.1016/j.cpr.2022.102235 36459876

[CR57] Muniandy, M., Richdale, A. L., & Lawson, L. P. (2023). Stress and well-being in autistic adults: Exploring the moderating role of coping. *Autism Research*, *16*(11), 2220–2233. 10.1002/aur.3028. 37698532 10.1002/aur.3028

[CR58] Newell, V., Phillips, L., Jones, C., Townsend, E., Richards, C., & Cassidy, S. (2023). A systematic review and meta-analysis of suicidality in autistic and possibly autistic people without co-occurring intellectual disability. *Molecular Autism*, *14*(1), 12. 10.1186/s13229-023-00544-7. 36922899 10.1186/s13229-023-00544-7 PMC10018918

[CR59] Nock, M. K., Hwang, I., Sampson, N., Kessler, R. C., Angermeyer, M., Beautrais, A., Borges, G., Bromet, E., Bruffaerts, R., de Girolamo, G., de Graaf, R., Florescu, S., Gureje, O., Haro, J. M., Hu, C., Huang, Y., Karam, E. G., Kawakami, N., Kovess, V., & Williams, D. R. (2009). Cross-national analysis of the associations among mental disorders and suicidal behavior: Findings from the WHO World Mental Health Surveys. *PLoS Medicine*, *6*(8), e1000123. 10.1371/journal.pmed.1000123. 19668361 10.1371/journal.pmed.1000123 PMC2717212

[CR60] Osman, A., Bagge, C. L., Gutierrez, P. M., Konick, L. C., Kopper, B. A., & Barrios, F. X. (2001). The suicidal behaviors Questionnaire-revised (SBQ-R): Validation with clinical and nonclinical samples. *Assessment*, *8*(4), 443–454. 10.1177/107319110100800409. 11785588 10.1177/107319110100800409

[CR61] Pelton, M. K., & Cassidy, S. A. (2017). Are autistic traits associated with suicidality? A test of the interpersonal-psychological theory of suicide in a non-clinical young adult sample. *Autism Research*, *10*(11), 1891–1904. 10.1002/aur.1828. 28685996 10.1002/aur.1828 PMC5697632

[CR62] Pelton, M. K., Crawford, H., Robertson, A. E., Rodgers, J., Baron-Cohen, S., & Cassidy, S. (2020). Understanding suicide risk in autistic adults: Comparing the interpersonal theory of suicide in autistic and non-autistic samples. *Journal of Autism and Developmental Disorders*, *50*(10), 3620–3637. 10.1007/s10803-020-04393-8. 32125567 10.1007/s10803-020-04393-8

[CR63] Pfaff, J. J., & Almeida, O. P. (2004). Identifying suicidal ideation among older adults in a general practice setting. *Journal of Affective Disorders*, *83*(1), 73–77. 10.1016/j.jad.2004.03.006. 15546648 10.1016/j.jad.2004.03.006

[CR64] Pratt, L. A., & Brody, D. J. (2014). Implications of two-stage depression screening for identifying persons with thoughts of self-harm. *General Hospital Psychiatry*, *36*(1), 119–123. 10.1016/j.genhosppsych.2013.09.007. 24183490 10.1016/j.genhosppsych.2013.09.007 PMC5942185

[CR65] Qualtrics. (2017). *Qualtrics (version 2.16) [computer program]*. In Qualtrics.

[CR66] Radoeva, P. D., Ballinger, K., Ho, T., Webb, S. J., & Stobbe, G. A. (2022). Risk and protective factors associated with depressive symptoms among autistic adults. *Journal of Autism and Developmental Disorders*, *52*(6), 2819–2824. 10.1007/s10803-021-05085-7. 34189682 10.1007/s10803-021-05085-7 PMC10027384

[CR67] Richards, G., Kenny, R., Griffiths, S., Allison, C., Mosse, D., Holt, R., O’Connor, R. C., Cassidy, S., & Baron-Cohen, S. (2019). Autistic traits in adults who have attempted suicide. *Molecular Autism*, *10*(1), 26. 10.1186/s13229-019-0274-4. 31198526 10.1186/s13229-019-0274-4 PMC6555998

[CR69] Richdale, A. L., Haschek, A., Chetcuti, L., & Lawson, L. (2022). *Longitudinal study of Australian school leavers with autism (SASLA): Final Report* https://www.autismcrc.com.au/knowledge-centre/reports/SASLA.

[CR68] Richdale, A. L., Chetcuti, L., Hayward, S. M., Abdullahi, I., Morris, E. M. J., & Lawson, L. P. (2023a). The impact of sleep quality, fatigue and social well-being on depressive symptomatology in autistic older adolescents and young adults. *Autism Research*, *16*(4), 817–830. 10.1002/aur.2899. 36772969 10.1002/aur.2899

[CR70] Richdale, A. L., Lawson, L. P., Chalmers, A., Uljarević, M., Morris, E. M. J., Arnold, S. R. C., & Trollor, J. N. (2023b). Pathways to anxiety and depression in autistic adolescents and adults. *Depression and Anxiety*, *2023*, 5575932. 10.1155/2023/5575932. 10.1155/2023/5575932 PMC11921850 40224589

[CR71] Ryff, C. D., & Keyes, C. L. (1995). The structure of psychological well-being revisited. *Journal of Personality and Social Psychology*, *69*(4), 719–727. 10.1037/0022-3514.69.4.719. 7473027 10.1037//0022-3514.69.4.719

[CR72] Sharaf, A. Y., Thompson, E. A., & Walsh, E. (2009). Protective effects of self-esteem and family support on suicide risk behaviors among at-risk adolescents. *Journal of Child and Adolescent Psychiatric Nursing*, *22*(3), 160–168. 10.1111/j.1744-6171.2009.00194.x. 19702970 10.1111/j.1744-6171.2009.00194.x

[CR73] Siegmann, P., Teismann, T., Fritsch, N., Forkmann, T., Glaesmer, H., Zhang, X. C., Brailovskaia, J., & Margraf, J. (2018). Resilience to suicide ideation: A cross-cultural test of the buffering hypothesis. *Clin Psychology & Psychotherapy*, *25*(1), e1–e9. 10.1002/cpp.2118. 10.1002/cpp.2118 28853242

[CR74] Simon, G. E., Rutter, C. M., Peterson, D., Oliver, M., Whiteside, U., Operskalski, B., & Ludman, E. J. (2013). Do PHQ depression questionnaires completed during outpatient visits predict subsequent suicide attempt or suicide death? *Psychiatric Services*, *64*, 1195–1202. 10.1176/appi.ps.201200587. 24036589 10.1176/appi.ps.201200587 PMC4086215

[CR75] South, M., Beck, J. S., Lundwall, R., Christensen, M., Cutrer, E. A., Gabrielsen, T. P., Cox, J. C., & Lundwall, R. A. (2020). Unrelenting depression and suicidality in women with autistic traits. *Journal of Autism and Developmental Disorders*, *50*(10), 3606–3619. 10.1007/s10803-019-04324-2. 31820343 10.1007/s10803-019-04324-2

[CR76] Tabachnick, B. G., & Fidell, L. S. (2007). *Using Multivariate statistics* (5th ed.). Allyn & Bacon.

[CR77] Teismann, T., Brailovskaia, J., Siegmann, P., Nyhuis, P., Wolter, M., & Willutzki, U. (2018a). Dual factor model of mental health: Co-occurrence of positive mental health and suicide ideation in inpatients and outpatients. *Psychiatry Research*, *260*, 343–345. 10.1016/j.psychres.2017.11.085. 29232575 10.1016/j.psychres.2017.11.085

[CR78] Teismann, T., Forkmann, T., Brailovskaia, J., Siegmann, P., Glaesmer, H., & Margraf, J. (2018b). Positive mental health moderates the association between depression and suicide ideation: A longitudinal study. *International Journal of Clinical and Health Psychology*, *18*(1), 1–7. 10.1016/j.ijchp.2017.08.001. 30487904 10.1016/j.ijchp.2017.08.001 PMC6220923

[CR79] Tennant, R., Hiller, L., Fishwick, R., Platt, S., Joseph, S., Weich, S., Parkinson, J., Secker, J., & Stewart-Brown, S. (2007). The Warwick-Edinburgh Mental Well-being scale (WEMWBS): Development and UK validation. *Health and Quality of Life Outcomes*, *5*, 63–63. 10.1186/1477-7525-5-63. 18042300 10.1186/1477-7525-5-63 PMC2222612

[CR80] Uljarević, M., Richdale, A. L., McConachie, H., Hedley, D., Cai, R. Y., Merrick, H., Parr, J. R., & Le Couteur, A. (2017). The Hospital anxiety and Depression scale: Factor structure and psychometric properties in older adolescents and young adults with autism spectrum disorder. *Autism Research*, *11*(2), 258–269. 10.1002/aur.1872. 28922575 10.1002/aur.1872

[CR2] van Agelink, J. A., Lever, A. G., & Geurts, H. M. (2019). Negatively phrased items of the Autism Spectrum quotient function differently for groups with and without autism. *Autism*, *23*(7), 1752–1764. 10.1177/1362361319828361. 30818972 10.1177/1362361319828361 PMC6728748

[CR81] van Heijst, B. F., Deserno, M. K., Rhebergen, D., & Geurts, H. M. (2020). Autism and depression are connected: A report of two complimentary network studies. *Autism*, *24*(3), 680–692. 10.1177/1362361319872373. 31709804 10.1177/1362361319872373 PMC7168804

[CR82] Waterman, A. S. (1993). Two conceptions of happiness: Contrasts of personal expressiveness (eudaimonia) and hedonic enjoyment. *Journal of Personality and Social Psychology*, *64*(4), 678–691. 10.1037/0022-3514.64.4.678.

[CR83] Westerhof, G. J., & Keyes, C. L. (2010). Mental illness and mental health: The two continua model across the lifespan. *Journal of Adult Development*, *17*(2), 110–119. 10.1007/s10804-009-9082-y. 20502508 10.1007/s10804-009-9082-y PMC2866965

[CR84] Wilhelm, K., Wedgwood, L., Parker, G., Geerligs, L., & Hadzi-Pavlovic, D. (2010). Predicting mental health and well-being in adulthood. *The Journal of Nervous and Mental Disease*, *198*(2), 85–90. 10.1097/NMD.0b013e3181cc41dd. 20145481 10.1097/NMD.0b013e3181cc41dd

[CR85] Zigmond, A. S., & Snaith, R. P. (1983). The hospital anxiety and depression scale. *Acta Psychiatrica Scandinavica. 67*(6), 361–370. 10.1111/j.1600-0447.1983.tb09716.x. 10.1111/j.1600-0447.1983.tb09716.x 6880820

