# Supplementary material for: Examination of the Potential Moderating Role of Psychological Wellbeing in the Relationship Between Depression and Thoughts of Self-Harm in Autistic Adolescents and Adults: A Two-Year Longitudinal Study
Source: J Autism Dev Disord. 2024 Jul 30;55(11):3928–40. doi: 10.1007/s10803-024-06489-x (PMC12575448; doi:10.1007/s10803-024-06489-x)
Supplement: Supplementary file 1 — Supplementary Table S1 [file 10803_2024_6489_MOESM1_ESM.pdf]

Supplementary Table S1

Mean (SD), and 5000 Sample Bootstrapped Comparisons for Included (n = 209) and Excluded (n = 319) Participants on Study Variables (T1)

Variable	Included			Excluded			Comparison Statistics [BCa 95% CI][1]
	n	*M (%)*	*SD*	*n*	*M (%)*	*SD*	
Gender							
Male	99	(47.4)	–	102	(32)	–	
Female	101	(48.3)	–	196	(61.4)	–	$\chi^2(2) = 12.874$, $p = .002$, Cramer's $V = .156$ [.066, .258]
Non-binary	9	(4.3)	–	21	(6.6)	–	
Age	209	34.13	15.381	318	38.84	12.714	$t(525) = 3.828$, $p <.001$, [2.21, 7.39], $d = .341$ [.165, .516]
W-ADL	162	30.14	4.840	256	31.63	3.728	$t(416) = 3.556$, $p = .001$, [.636, 2.33], $d = .357$ [.159, .555]
AQ-Short	206	83.89	12.927	299	85.48	12.848	$t(503) = 1.729$, $p = .085$, [–.296, 3.88], $d = .156$ [–.021, .333]
WEMWBS	204	40.55	10.017	276	40.75	10.172	$t(478) = .210$, $p = .840$, [–1.633, 1.982], $d = .019$ [–.162, .200]
HADS-D (SASLA)	67	5.84	3.333	15	5.53	3.563	$t(80) = –.314$, $p = .754$, [–2.303, 1.587], $d = –.090$ [–.649, .471]
PHQ-8 (ALSAA)	121	9.64	6.353	239	10.55	6.324	$t(358) = 1.290$, $p =.198$, [–.505, 2.256], $d = .144$ [–.075, .363]
PHQ-8 (SASLA/ALSAA)	197	9.04	6.252	263	10.26	6.339	$t(458) = 2.058$, $p =.038$, [.057, 2.380], $d = .194$ [.009, .379]
Thoughts of Self-harm	201	.56	.876	268	0.64	0.956	$t(467) = .939$, $p =$ .348 [-.090, .251], $d = .088$ [–.095, .271]

Note. [1]Bonferroni adjusted *p*-value for 8 tests = .00625. W-ADL: Waisman Activities of Daily Living (Maenner et al., 2013); AQ-Short: Autism Quotient, Short (Hoekstra et al., 2011); WEMWBS: Warwick-Edinburgh Mental Wellbeing Scale (Tennant et al., 2007); HADS-D: Hospital Anxiety and Depression Scale (SASLA; Zigmond & Snaith, 1983); PHQ-8: Patient Health Questionnaire, 8 item (Kroenke et al., 2009); Thoughts of Self-harm: PHQ, item 9 (Kroenke & Spitzer, 2002; Kroenke et al., 2001).
