# Supplementary material for: Examination of the Potential Moderating Role of Psychological Wellbeing in the Relationship Between Depression and Thoughts of Self-Harm in Autistic Adolescents and Adults: A Two-Year Longitudinal Study
Source: J Autism Dev Disord. 2024 Jul 30;55(11):3928–40. doi: 10.1007/s10803-024-06489-x (PMC12575448; doi:10.1007/s10803-024-06489-x)
Supplement: Supplementary file 2 — Supplementary Table S2 [file 10803_2024_6489_MOESM2_ESM.pdf]

Supplementary Table S2

Bootstrapped Hierarchical Linear Regression Models with T1 Variables and Group (SASLA, ALSAA) Predicting Thoughts of Self-harm at T2

	b	***SEB***	***β***	***p*-value**[b]	**BCa 95% CI**[c]
**Constant**	–0.176	0.169	–	.391	[–.492, .167]
Group	0.095	0.102	0.048	**.439**	[–.105, .285]
Thoughts of Self-harm (T1)	0.500	0.075	0.513	**<.001**	**[.360, .655]**
Autistic Traits[a]	0.138	0.055	0.143	**.027**	**[.038, .243]**
Wellbeing[a]	0.096	0.071	0.097	.195	[–.042, .232]
Depression[a]	0.265	0.079	0.262	**<.001**	**[.113, .424]**
Depression × Wellbeing[a]	0.114	0.063	0.113	.064	[–.010, .236]
Model	R^2 = 0.402, $F(6, 200)$ = 21.756, $p < .001$				

Note. [a]*z*-score used in analysis. [b]5000 samples bootstrapped *p*-value. [c]BCa 95% confidence intervals that do not cross zero are bolded.
