# Supplementary material for: Examination of the Potential Moderating Role of Psychological Wellbeing in the Relationship Between Depression and Thoughts of Self-Harm in Autistic Adolescents and Adults: A Two-Year Longitudinal Study
Source: J Autism Dev Disord. 2024 Jul 30;55(11):3928–40. doi: 10.1007/s10803-024-06489-x (PMC12575448; doi:10.1007/s10803-024-06489-x)
Supplement: Supplementary file 3 — Supplementary Table S3 [file 10803_2024_6489_MOESM3_ESM.pdf]

Supplementary Table S3

Bootstrapped Linear Regression Model with T1 Variables Predicting Thoughts of Self-harm (T2) utilizing PHQ8 (T1) Depression (SASLA, ALSAA Samples)

	b	***SEB***	***β***	***p*-value**[b]	**BCa 95% CI**[c]
**Constant**	0.006	0.063	–	.928	[–.122, .144]
Thoughts of Self-harm (T1)	0.467	0.067	0.487	**<.001**	**[.306, .642]**
Autistic Traits[a]	0.112	0.059	0.118	.051	**[.002, .225]**
Wellbeing[a]	0.065	0.075	0.068	.370	[–.078, .217]
Depression[a]	0.197	0.080	0.207	**.016**	**[.037, .363]**
Depression × Wellbeing[a]	0.063	0.049	0.079	.204	[–.039, .164]
Model	$R^2 = 0.362$, $F(5, 196) = 21.661$, $p < .001$				

Note. [a]*z*-score used in analysis. [b]5000 samples bootstrapped *p*-value. [c]BCa 95% confidence intervals that do not cross zero are bolded.
